# scFv Antibody: Principles and Clinical Application

**DOI:** 10.1155/2012/980250

**Published:** 2012-03-15

**Authors:** Zuhaida Asra Ahmad, Swee Keong Yeap, Abdul Manaf Ali, Wan Yong Ho, Noorjahan Banu Mohamed Alitheen, Muhajir Hamid

**Affiliations:** ^1^Department of Cell and Molecular Biology, Faculty of Biotechnology and Biomolecular Sciences, Universiti Putra Malaysia, Selangor, 43400 Serdang, Malaysia; ^2^Institute of Bioscience, Universiti Putra Malaysia, Selangor, 43400 Serdang, Malaysia; ^3^Faculty of Agriculture and Biotechnology, Universiti Sultan Zainal Abidin, Kampus Kota, Jalan Sultan Mahmud, 20400 Kuala Terengganu, Malaysia; ^4^Department of Bioprocess Technology, Faculty of Biotechnology and Biomolecular Sciences, Universiti Putra Malaysia, Selangor, 43400 Serdang, Malaysia

## Abstract

To date, generation of single-chain fragment variable (scFv) has become an established technique used to produce a completely functional antigen-binding fragment in bacterial systems. The advances in antibody engineering have now facilitated a more efficient and generally applicable method to produce Fv fragments. Basically, scFv antibodies produced from phage display can be genetically fused to the marker proteins, such as fluorescent proteins or alkaline phosphatase. These bifunctional proteins having both antigen-binding capacity and marker activity can be obtained from transformed bacteria and used for one-step immunodetection of biological agents. Alternatively, antibody fragments could also be applied in the construction of immunotoxins, therapeutic gene delivery, and anticancer intrabodies for therapeutic purposes. This paper provides an overview of the current studies on the principle, generation, and application of scFv. The potential of scFv in breast cancer research is also discussed in this paper.

## 1. Introduction

Antibodies are a modular defense system that identify and neutralize foreign objects like bacteria and viruses. Each of them could recognize a specific antigen unique to its target as they possess the antigen-binding sites, a paratope (a structure analogous to a lock) located at the upper tips of the “Y” shaped antibody molecules. This paratope is specific for one particular epitope (analogous to a key), displayed on the particular antigen, allowing these two structures to specifically bind together. Thus, this mechanism could allow an antibody to tag a microbe as well as an infected cell to be attacked by other parts of the immune system and also to directly neutralize its target [1].

## 2. Recombinant Antibody Technology

Being a major part of the immune system, antibodies represent a powerful weapon system in defending our body against non-self-agents. But, to interact with as many foreign structures as possible, an enormous number of different molecules, bearing different specificities, are needed. This diversity can be produced through somatic recombinant and hypermutagenesis of a set of variant genes [2].

During the past decade, advances in recombinant antibody technology have greatly facilitated the genetic manipulation of antibody fragments [4, 5]. The genetic manipulation of recombinant antibodies thus improved our understanding about the structure and functional organization of immunoglobulins. Further, these advances have led the development of a large variety of engineered antibody molecules for research, diagnosis, and therapy with specificities out of reach of conventional antibody technology. Once cloned, it is then possible to increase the affinity and specificity of antigen binding by mimicking somatic hypermutation during an immune response [6]. It may even be possible to replace the existing practices of animal immunization and hybridoma development through a bacterial system capable of synthesizing and expressing practically unlimited quantities of antibodies to almost any antigen.

Since 1975, Kohler and Milstein have introduced the hybridoma technology which enabled a defined specificity of monoclonal antibodies to be produced in consistent quality as well as in large quantities in the laboratory. Since then, monoclonal antibodies (mAbs) have been favored as they can be produced in unlimited quantities to practically bind to any antigen and are more easily standardized [7, 8]. Besides monoclonal antibodies, hybridoma cells that are successfully produced could then serve as a starting material in the generation of Fab, or Fv fragments in lymphoid or nonlymphoid cells [9]. Monoclonal antibodies face several difficulties, as they are almost exclusively murine in origin thus could create human anti-mouse antibody (HAMA) when introduced to human therefore limits their clinical applications [7, 8]. Added to this, monoclonal antibody producing technology is very laborious and time consuming. Furthermore, small mammals like mice do not always provide the high-affinity antibody response to particular antigen needed for sensitive assay development [10]. These limitations of traditional techniques have led several research groups to investigate the use of phage display in producing scFv antibodies. 

The first study in producing recombinant antibodies in bacteria was hampered due to improper folding and aggregation of the polypeptide in the bacterial cytoplasm [4, 11]. In order to overcome these problems, Skerra and Plückthun [12] have introduced a one-step-forward technology whereby only parts of the antibody molecule (Fab or Fv fragments) are used for expression purposes. The breakthrough for competent *E. coli *expression of antibody fragments yet was brought by introducing several types of vectors (e.g., phagemid) to be used in the recombinant antibody construction. These vectors could offer soluble antibody secretion directly into the periplasm space by means of its oxidizing environment that contributes for the correct formation of disulphide bonds between the antibody domains [12, 13].

Recently, the technology has been improved through recombinant DNA technology and antibody engineering whereby antibody genes can now be cloned and expressed successfully as a fragment in bacteria [12], on mammalian cell and yeast [15], plant [16], and also insect cells [17]. One advantage of this new technology is that they could retain the intact antigen binding site (paratope) while reducing the size of the antibody molecule. In comparison to the parental antibody, these minimized antibodies have several advantages in clinical practices including better tumor penetration, more rapid blood clearance, and lower retention times in nontarget tissue and also to reduced immunogenicity. It also could lead to the expression of the functional antibody and their fusion in bacteria and also allow their display on a filamentous phage. In addition, the combination of small antibody molecule together with the efficient microbial production systems can finally lead to the production of a homogenous protein in sufficient amounts for diagnostic and therapeutic purposes as well as in structural studies [18–20].

Thus, with the latest technology, a wide variety of genetically engineered antibodies have been produced as reviewed by a number of researchers [21–23] including Fab fragments [24], Fv fragments in which the *V* domains are held together by noncovalent forces [12] and so-called single-chain fragment variable or scFv (single chain fragment variable) antibodies in which the genes of *V*_*H*_ and *V*_*L*_ dare joined together with a short flexible peptide linker or with disulfide bond [25]. Alternatively, the minimized antibodies molecule could also serve as a building block for the construction of new recombinant proteins for different purposes. For instance, antibody with the same affinity have been successfully fused to construct bivalent antibodies [26] or multivalent antibodies [27, 28] and two antibody fragments with different specificities have been fused to construct bispecific antibodies [29].

## 3. Single-Chain Fragment Variable (scFv)

**Figure 1:**
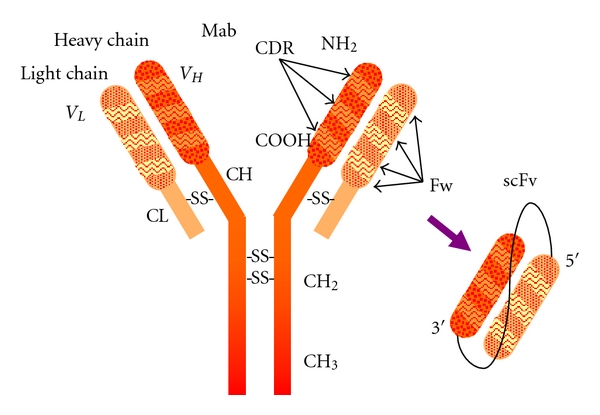
Antibody model showing subunit composition and domain distribution along the polypeptide chains. Single-chain fragment variable (scFv) antibody generated by recombinant antibody technology appears in the shaded area.

Fv fragment is the smallest unit of immunoglobulin molecule with function in antigen-binding activities. An antibody in scFv (single chain fragment variable) (Figure 1) format consists of variable regions of heavy (*V*_*H*_) and light (*V*_*L*_) chains, which are joined together by a flexible peptide linker that can be easily expressed in functional form in *E. coli*, allowing protein engineering to improve the properties of scFv (single chain fragment variable) such as increase of affinity and alteration of specificity [30].

The length of the flexible DNA linker used to link both of the *V* domains is critical in yielding the correct folding of the polypeptide chain. Previously, it has been estimated that the peptide linker must span 3.5 nm (35 Å) between the carboxy terminus of the variable domain and the amino terminus of the other domain without affecting the ability of the domains to fold and form an intact antigen-binding site [31]. In addition to the linker peptides designed *de novo*, peptide sequences derived from known protein structure have been applied to provide a compatible length and conformational in bridging the variable domains of a scFv (single-chain fragment variable) without serious steric interference [32–34]. Apart from the length of the linker, their amino acid composition also plays an important role in the design of a viable linker peptide. They must have a hydrophilic sequence in order to avoid intercalation of the peptide within or between the variable domains throughout the protein folding [35]. Nowadays, the most extensively used designs have sequences comprising stretches of Gly and Ser residues which meant for flexibility and or together with the charged residues such as Glu and Lys interspersed to enhance the solubility [36].

## 4. The Generation of scFv (Single-Chain Fragment Variable) Antibodies

scFv (single chain fragment variable) antibodies have been constructed mainly from hybridoma [16, 37–39], spleen cells from immunized mice [40–42], and B lymphocytes from human [43–45]. scFv (single chain fragment variable) is a noncovalent heterodimer comprised of the *V*_*H*_ and *V*_*L*_ domains [12] in which can then be used in the construction of recombinant scFv (single chain fragment variable) antibody. In order to attain them, mRNA is first isolated from hybridoma (or also from the spleen, lymph cells, and bone morrow) followed by reverse transcribed into cDNA to serve as a template for antibody genes amplification (PCR). With this method, large libraries with a diverse range of antibody *V*_*H*_ and *V*_*L*_ genes could be created [43]. One successful approach to recombinant antibody production has been developed by McCafferty and coworkers in which they utilized the phage recombinants that are displaying antibody at their tips together with the new techniques of affinity selection, called biopanning step. The work by McCafferty et al. [14] thus has opened the opportunity for* in vitro* selection of scFv (single chain fragment variable) from large libraries of variable domains circumventing the traditional hybridoma method.

In the scFv (single-chain fragment variable) construction, the order of the domains can be either *V*_*H*_-linker-*V*_*L*_ or *V*_*L*_-linker-*V*_*H*_ and both orientations have been applied [46–48]. Even though Luo et al. [49] have shown that the expression of scFv (single-chain fragment variable) of *Pichia pastoris *system is *V*_*L*_-linker-*V*_*H*_ orientation-dependent; most of the scFv (single-chain fragment variable) are constructed in a *V*_*H*_-linker-*V*_*L*_ orientation. One of the most popular methods used is through PCR assembly [40, 43, 50] which was first described by Horton et al. [51]. In this method, it allows the *V* domains of antibody to be cloned without any prior information about the nucleic acid as well as amino acid sequence of the particular antibody [52]. Moreover, the *V* domains of antibody can be combined by *in vitro *recombination directly after the PCR of *V*_*H*_ and *V*_*L*_ genes into plasmid [38, 53] or phagemid [54]. Alternatively, scFv (single-chain fragment variable) can also be constructed with sequential cloning [55, 56] or combinatorial infection [57].

Numerous scFv (single-chain fragment variable) have been constructed against hapten [55], protein [46, 58], carbohydrate [59, 60], receptor [16], tumor antigen [44, 61], and viruses [47]. All these scFv (single chain fragment variable) have good potential for use in many fields such as medical therapies and diagnostic applications.

## 5. The Expression of Antibody Fragments (scFv)

To date, antibody fragments (scFv) have been successfully isolated and displayed as fragments in various expression systems such as mammalian cell and yeast [15], plant [16], and also insect cells [17]. scFv (single-chain fragment variable) antibody can be expressed as correctly folded and directly active proteins or as aggregates requiring *in vitro *refolding to become active. Depending on the expression system, it varies in their ability to fold and secrete the scFv (single-chain fragment variable) proteins. There are some general regulations to consider on the design of vectors and expression system used with the different hosts and each of this host has advantages and disadvantages for the production of active scFv (single-chain fragment variable) antibody [62]. Nevertheless, the bacterial expression system is most often applied for the production of scFv (single-chain fragment variable) antibody fragments compared to the various expression strategies available.

Recent progress in the perceptive of both genetics and biochemistry of *E. coli *makes this organism a precious tool as an expression host [63]. Moreover, scFv (single-chain fragment variable) antibody with good folding properties can be expressed economically by using this organism compared to whole antibodies with complicated folding and glycosylation which require slow and expensive cell culture techniques. Apart from that, it also could promise high throughput of recombinant proteins and can be used in multi-plexed cloning, expression, and purification of proteins for structural genomics [64]. Further, this production system is well-studied physiology, genetics, and availability of advanced genetic tools [63, 65, 66], rapid growth, a very high yields up to 10–30% of total cellular protein, a simple way to conduct within a standard molecular biology laboratory, low cost, and the capability to multiplex both expression screening [67] together with the protein production [68].

There are a number of different strategies used to express the recombinant antibody fragments in *E. coli as* reviewed by investigators [12, 69]. One way is to express scFv (single-chain fragment variable) antibody directly in the cytoplasm of *E. coli *without using a signal peptide [32, 70]. As a result, the polypeptides are greatly expressed in the reducing environment of bacterial cytoplasm followed by the formation of insoluble aggregates called inclusion bodies. For that reason, the inclusion bodies must be renatured *in vitro *to improve the correct folding of functional protein by means of appropriate rearrangement of the disulphide bonds [71]. To overcome this problem, a signal peptide is used to direct secretion of the scFv (single-chain fragment variable) antibody into the periplasmic space that lies between inner and outer membranes of gram-negative bacteria [12, 13]. According to the Baneyx [63], this periplasmic space is identified to contain protein such as chaperones and disulphide isomerases, which assist the proper folding of recombinant proteins. Generally, the Fab fragment produced in *E. coli* is based on the expression of a discistronic operon unit where both of the genes (L and Fd) are controlled by the same promoter and thus allowing synthesis of both chains in an equal amount. During translocation via the inner membrane into the oxidizing environment of the periplasm, the signal peptides attached at the N-terminus are sliced off permitting the chains to fold and assemble and thus lead to the formation of both of the intra- and interdomain sulphide. Until now, periplasmic expression has been extensively employed in the production of various specific scFv (single-chain fragment variable) antibodies. But, in some cases, antibodies are observed as insoluble material since their efficiency and folding are depending on the individual protein [34].

At present, studies on the factor to improve both phage display as well as periplasmic folding of scFv (single-chain fragment variable) fragments have led to the discovery of accessory proteins for decrease aggregation and together with facilitated folding of the scFv (single-chain fragment variable) antibody fragments [72–75]. An antibody in scFv (single-chain fragment variable) format possesses the hydrophobic patches that are buried in a hydrophobic variable or constant interface. Once they are exposed to the solvent, it possibly will promote aggregation during protein folding. By replacement of the hydrophobic residues by hydrophilic ones has been shown to improve the *in vivo *folding properties of the protein [76].

Alternatively, disulphide bonds of antibodies are also contributing significantly in the stability of the structure and yet make them conserved in evolution. For instance, there are studies that demonstrated that the replacement of the disulphide forming cysteines with other amino acids enabled to produce the same active and correctly folded antibody fragments [77].

## 6. Phage Display

Beginning in 1985, Smith [78] was first to reveal that foreign DNA fragments can be fused to the gene encoded for pIII coat protein of a nonlytic filamentous phage and expressed as a fusion protein on the virion surface without disturbing the infectivity of the phage. A few years later, McCafferty et al. [14] in their study have successfully demonstrated that a scFv (single-chain fragment variable) fragment can be displayed on the phage surfaces as a functional protein which retains an active antigen-binding domain capability. Therefore, this technology could allow rare clones to be screened and isolated from a large population of phage using any desirable antigen [79]. Since these first applications, the technology has now been widely used to other antibody molecules including Fab fragments [80], disulphide-stabilized Fv fragments [48], and diabodies [81].

There are several types of libraries: phage display libraries (immune, naïve, and synthetic) and ribosomal display libraries. But, each type of library has its limitations and is suitable for different purposes depending on the nature of antigen panned during selection procedure together with the affinity and number of antibodies expected. Based on the source of antibody genes, scFv (single-chain fragment variable), libraries can be divided into three different categories; immune, naïve, and synthetic. Firstly, immune libraries which have been constructed from variable domains of antibody genes of B cells derived from different kinds of immunized animals like mice [82], camel [83], sheep [84], and also humans [85]. In these libraries, the enriched antibodies are biased towards antigen that is used for immunization. Due to the *in vivo *affinity maturation (by the hosts' immune system) of the antibodies, this approach always results in a great range of higher affinities of isolated binders as well as the number of binders specific towards the antigen but yet, the construction of the new library for each antigen is needed.

Secondly, naïve libraries, derived from nonimmunized donors of B cells that have been constructed from a pool of *V*-genes of IgM mRNA [86, 87]. Unlike immune libraries, these libraries are not biased towards any antigen and they are utilized in the selection of antibodies against different specificity without the need to construct a new library for each antigen. Therefore, they are particularly useful for the production of antibody fragments which are difficult to generate using hybridoma technology especially against nonimmunogenic or toxic antigens. While the affinities of mAbs produced very much depending on the library size (between 10^9^-10^10^ members), even affinities below 1 nM were reported [87]. Generally, the affinities of scFv (single-chain fragment variable) isolated from small naive libraries are lower compared to those isolated from larger libraries [86–89]. For that reason, the size of the library is important for the selection of high-affinity fragments as well as to determine the success rate of selection of phage against a large repertoire of different antigens [86, 87].

Thirdly, the synthetic libraries which also derive from nonimmune sources as their ranges were prepared synthetically by combining germ line gene sequences together with randomized complementary determining regions (CDRs) that are responsible for antigen binding [89, 90]. The majority of synthetic human antibody libraries produced focused on randomizing the CDR3 regions, which are usually most diverse and essentially responsible for antigen binding. Therefore, these libraries are extensively chosen to yield high affinity of monoclonal antibodies. There are a number of large semisynthetic libraries available now; one of the largest called the Griffin-1 library contain human scFv (single-chain fragment variable) fragments (H Griffin, MRC, Cambridge, UK, unpublished data). This study was performed by Griffiths et al. [89] by means of recloning synthetic heavy and light chain variable regions from a 2-lox Fd phage library vector into the pHEN2 phagemid vector. There are also different studies that have been developed to construct fully synthetic scFv (single-chain fragment variable) libraries based on single or multiple variable domains used as scaffold and CDRs of varying composition and length [90, 91].

Other than phage display, there are also libraries constructed using ribosomal display technology which utilized *in vitro* method to isolate scFv (single-chain fragment variable) directly without involving phage and bacteria organisms. Firstly, the DNA library of scFv (single-chain fragment variable) is transcribed and translated *in vitro* to create the complex of linked mRNA-ribosome-scFv (single-chain fragment variable) protein which is used for selection on immobilized antigen. The mRNAs that are specifically bound to the antigen then eluted, followed by reverse transcribtion and finally the enriched regenerated DNA pool then employed for the next round of selection [92, 93]. Since it was a relatively new technology, and thus not fully utilized, yet it has the potential to further simplify and shorten during scFv (single-chain fragment variable) selection. These techniques, however, could provide a great tool for the generation of an unlimited number of monoclonal antibodies by means of in vitro as well as in vivo procedure which are biased towards antigen that is interested.

**Figure 2:**
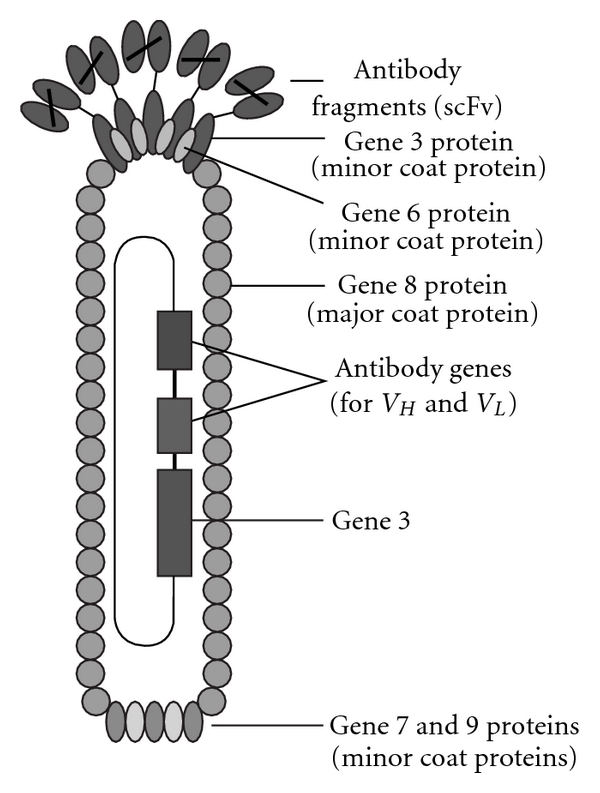
Structure of a filamentous phage displaying scFv fragments on its surface [14].

In order to express the antibody fragments on the surface of the filamentous bacteriophage, the immunoglobulin genes can be fused either to the VIII gene [94] or the pIII gene (Figure 2) [14]. The single-stranded DNA genome of the filamentous phage is coated with approximately 2700 copies of the major coat protein (pVIII) and can be fused together with antibody to result in multivalent display of the fragment. While three to five copies of the minor coat protein pIII are located on the tip of the phage, which is responsible for attaching the phage to its bacterial host during infection. As reviewed by researchers [95, 96], the most widely used display format is derived from the use of the phagemid vector and the pIII display, which allows high frequency of monovalent display and therefore is preferred in affinity selection.

The ultimate aim of phage display is the selection of phage that can bind to the target antigen of interest with high affinity from a huge number of nonspecific phage clones. This is achieved by multiple rounds of phage binding to the antigen, washing to remove the unbound phage followed by elution and retrieval of specifically bound phages. After each round, the eluted phages are amplified by the infection of *E. coli *for the following selection step. Soluble antibodies are produced in the final round of the bacteria then characterized. There are various types of selection strategy that can be used to attain specific antibodies [95, 96]. Generally, the procedures for selecting against purified antigens rely upon the phage binding affinity for the antigen of desire comparative to phage particles that do not bind, so that it require the antigen to be attached onto a solid support [40, 43, 89]. In the majority of this applications, selection has been carried out by incubating phage on antigen immobilized onto a solid support, such as Immunotubes [90, 97], microtiter plate wells [98], BIAcore sensor chips [97], or columns [98]. Or, the phage library can be incubated using biotinylated antigen in solution, followed by capture of the antigen-phage complex on streptavidin surface [89, 99]. With this approach, antibodies on the basis affinity can be selected by using an antigen concentration much less than the desired dissociation constant Kd [99–101]. In addition, by selecting an appropriate design for the selection procedure, antibodies can too be selected on the basis of kinetic properties [101, 102], improved specificity [103], or phage infectivity [104, 105].

In cases where pure antigen is not available, such as integral membrane protein, or the unknown antigen source (for novel markers on cells or tissues studies), specific antibodies must be isolated using more complex sources, such as whole cells [45, 106, 107] or tissue fragments [55]. In contrast, antibody fragments can also be raised against heterogeneous cell mixtures using fluorescence activated cells (FACs) sorting selection. Firstly, the phage library is incubated with the cells of interest while unbound phage is removed by washing thoroughly. Next, the cells of interest are labeled with a known fluorescence labeled Mab followed by cell sorting step and finally the sorted phages are eluted and amplified [108]. With this technique, unbound phage can be isolated by differential centrifugation of the cell-phage complex throughout an organic phase and the bound phage is recovered in the cell pellet [109].

In most applications, the binding characteristics of antibodies selected from phage libraries are frequently sufficient for further use in research applications, such as ELISA, Western blotting, or immunofluorescence [48]. However, the antibodies affinity or specificity selected from the libraries might be insufficient for diagnostic or therapeutic applications and therefore further improvement is needed. This problem could be improved by targeting mutagenesis to one or more of the CDRs or only a part of them followed by selection of high affinity antibodies [48, 110]. Despite mutagenesis, pairing heavy chain with a library of light chains (chain-shuffling), or vice versa, has been applied to increase the antibodies affinity which is in the nanomolar or picomolar range [42, 111].

In summary, phage display technology has been well developed in which we can generate new specific antibodies by circumventing immunization and consequently could facilitate the generation of antibodies that are difficult or impossible to gain through conventional methods, such as human antibodies and antibodies against self-antigens [86, 95, 112]. Moreover, during the selection procedures, phage display system can too be applied to isolate stable and well-folded proteins, catalyst, or peptide substrates [113].

## 7. Advantages of Phage-Displayed Single-Chain Variable Fragment (scFv)

There are several advantages of phage-displayed scFv (single-chain fragment variable) over monoclonal antibodies. Firstly, phages are more stable and can be stored up to several years at 4°C [114]. Secondly, they can be produced rapidly and inexpensively just by infecting the *E. coli *[115]. Thirdly, their genes can be easily manipulated and lastly they can be produced by circumventing hybridomas and immunization [43]. Also, higher affinity mutants of scFv (single-chain fragment variable) can be generated through site-directed mutagenesis which is much easier and simpler to be performed [50, 116]. In contrast to soluble scFv (single-chain fragment variable), phage-displayed scFv (single-chain fragment variable) can be used directly during mice immunization to produce anti-idiotypic antibodies without the use of adjuvants. This is because phage particles are also good immunogens [117].

## 8. Application of Single-Chain Fragment Variable (scFv) and Phage-Displayed Single-Chain Fragment Variable (scFv)

Since the introduction of hybridoma technology, the exquisite specificity of mAbs and their fusion proteins have been exploited for several applications in medicine, laboratory diagnosis, and research. Recent progress in antibody engineering together with the microbial expression systems could facilitate a powerful tool to design and produce new specificity and tailored antibodies at affordable prices as if for large-scale purposes. Moreover, tags or peptides attached to the antibodies could allow the purification of antibody fragments that can bring homogenous, well-defined, and active proteins.

### 8.1. Medical Application

In modern medicine, antibodies for therapeutics are well established as an important class of drugs. An antibody in scFv (single-chain fragment variable) format retained the complete antigen-binding capability, thus it could facilitate a potentially unique molecule to be used especially in cancer treatment [118]. In addition, the antibodies with exquisite specificity and affinity towards a specific target have also encouraged their development as delivery vehicles for agents such as radionuclides to target tissues, for radioimmunoimaging and radioimmunotherapy [119, 120].

The small antigen-binding molecule of scFv (single-chain fragment variable) antibodies could offer several advantages over a whole antibody molecule in therapeutic applications [20, 22]. The smaller fragments allow these molecules to penetrate more rapidly and evenly to tumors and other tissue in comparison to the whole antibodies [20, 121]. As these fragments have more rapid clearance from blood, they can be coupled with drugs and radionuclides in order to result in low exposure of the healthy tissue [19, 122, 123]. Besides that, they also found that there was no uptake of the scFv (single-chain fragment variable) by the kidney and they could efficiently localize to the tumors [124]. All these properties are important in cancer therapy. However, contrast idea has been proposed where the small molecular size of scFv (single-chain fragment variable) have the physiological disadvantage of rapid elimination from the body via kidney [125].

Generally, antibody fragments can be utilized for the preparation of immunotoxins, therapeutic gene delivery, and as anticancer intrabodies. Antibody fragments can be fused to a range of toxins such as cytotoxic proteins [126], radionuclides [127], or drugs [128]. Once fused, these immunotoxins could specifically deliver their agents towards cancer antigen-presenting cells. One example of immunotoxin that is currently generated for fatal neoplasm treatment was the fusion of a fully human anti-fAChR Fab-fragment to Pseudomonas exotoxin A to generate an anti-fAChR immunotoxin (scFv35-ETA) [129]. Following this, all the cancer cells are killed upon the immunotoxins being internalized. In addition to targeting the antigen, tumor-specific scFv (single-chain fragment variable) could too deliver promising therapeutic agents such as tumor necrosis factor (TNF) and to be fused to interleukin-2 (IL-2) or superantigen Staphylococcal Enterotoxin B- for T-cell-mediated eradication of tumors [130, 131]. Besides, Certolizumab Pegol that works as an immunoinhibitor to block the TNF-*α* has been recently approved by FDA to be used for the treatment of Crohn's disease and rheumatoid arthritis. It was found to be effective on various inflammatory diseases [132]. Efficacy of Certolizumab Pegol was supported by phase III clinical trial which was compared with the placebo [133].

#### 8.1.1. scFv (Single-Chain Fragment Variable) for Crohn's Disease and Rheumatoid Arthritis

Alternatively, antibody fragments could also play an important role in the gene therapy of cancer. It includes the therapeutic gene delivery applications where the viral and nonviral vector are used. These viral vectors are known to be very efficient in delivering therapeutic genes to target cell populations, but yet, their clinical use may be restricted by eliciting of immune response. While for nonviral vectors, they often lack their specificity in comparison to the viral vectors. Several studies were performed where scFv (single-chain fragment variable) were used in the development of specific viral and nonviral targeting vectors for therapeutic gene delivery. For instance, one study was performed by Kuroki et al. [134] in the development of a retroviral vector that displays anti-CEA-scFv against carcinoembryonic antigen (CEA). At the same time, this vector also brings together the therapeutic gene of nitric oxide synthase. As a result, this recombinant retrovirus can particularly bound, infect and kill the CEA-expressing cancer cells and finally verify cell-specific delivery of the therapeutic gene [134]. Other approach was carried out by Li et al. [135] in which they produced a nonviral vectors carrying anti-ErbB2 scFv (single-chain fragment variable) against a tumor-associated receptor that is overexpressed in most of human cancers (ErbB2). It was showed that these vectors are capable of delivering exogenous DNA into ErbB2 receptor expressing cells compared to cells that do not express these receptors.

Apart from immunotoxins and therapeutic gene delivery, there are also anticancer intrabodies which have been proved not only to be antiviral, but are also known as potent antitumor agents. Numerous studies have reported their ability in expressing within the cell, specifically binding and neutralizing a range of oncogenes together with signaling molecules. It has been found that this process is effective in decreasing tumor growth and in the potential admission of cells into the apoptotic cycle. Lately, there is anti-ErbB2 intrabody which was tested in phase I clinical trials. The treatment involved adenoviral-mediated gene therapy in conjunction with this intrabody and has proven to be possible in the treatment of ErbB2-overexpressing ovarian cancer [136]. Another study conducted by Strube and Chen [137] has proven that anticyclin E scFv (single-chain fragment variable) intrabody which are expressed in the nucleus of the breast cancer cell line could inhibit the growth of this cell line.

Up till now, several mAbs have been approved by the U.S. Food and Drug Administration (FDA) for the cancer treatment (Table 1). However, immunotherapy has been more successful against circulating cancer cells rather than solid tumors since they have greater cell accessibility. This is illustrated by the FDA approval of intact antibodies such as Rituxan, for the treatment of non-Hodgkin lymphoma and Campath and Mylotarg for the treatments of leukemia (Table 1) [138]. There only two monoclonal antibodies have been approved previously for the treatment of solid tumors which are Herceptin, for the treatment of breast carcinoma and PanoRex for colon cancer. Even though the mechanisms of action are still under investigation, Herceptin appears to make use of Fc receptors and angiogenesis [139], while Rituxan triggers apoptosis throughout receptor dimerization [140].

Since September 1998, Herceptin (Trastuzumab) has been approved for the treatment of HER2-positive metastatic breast cancer. This first humanized antibody ws then approved for the second time in November 2006 as part of a treatment regimen containing doxorubicin, cyclophosphamide, and paclitaxel, for the adjuvant treatment of patients with HER2-positive, node-positive breast cancer. Basically, Herceptin is designed to target and block the function of HER2 protein overexpression [141]. Research has shown that HER2-positive breast cancer is a more aggressive disease with greater chances of recurrence, a poorer prognosis, and a reduced chance of survival in comparison to HER2-negative breast cancer.

The most recent study was performed by Koido et al. [142] which suggest that tumor-cell lines (MCF-7) can be used as a fusion partner in the development of dendritic cell (DC) tumor fusion vaccine. Once successfully fused, they can be served as a vaccine for active immunotherapy or as stimulators to activate and expand T cells for adoptive immunotherapy applications. In separate study, McWhirter et al. [98] has produced cell-surface-associated proteins that overexpressed on B-cell chronic lymphocytic leukemia (CLL) which could serve as therapeutic antibody targets by encouraging a cytotoxic T-cell response.

### 8.2. Diagnostic Application

Another important application of the scFv (single-chain fragment variable) is as diagnostic reagents. During past years, tailor-made recombinant scFv (single-chain fragment variable) antibodies produced in bacteria have become potential alternatives to these “conventional” immunodiagnostic reagents [143]. The functionality of recombinant antibody fragments (scFv) as immunological reagents has been discovered in several different assay formats [144, 145].

In general, monoclonal antibody especially scFv (single-chain fragment variable) antibody for diagnostic purposed can bind to a variety of antigens such as haptens, proteins, and also whole pathogens, and they can as well be used in the enzyme-linked immunosorbent assay (ELISA) [146]. The detection of scFv (single-chain fragment variable) can be done using secondary antibody recognizing specific tag which already fused to the C- or N-terminus of scFv (single-chain fragment variable). A number of tags have been used previously, such as c-myc [147] or E-tag (Pharmacia). Due to improper folding of soluble scFv (single-chain fragment variable), they can be easily inactivated during being coated on the microtiter plates. This was caused by the lack of constant domains of heavy and light chains. In order to overcome this difficulty, (especially during the selection of scFv (single-chain fragment variable) from phage display libraries), scFv (single-chain fragment variable) in a phage format can be used in ELISA assay, which means that the scFv (single-chain fragment variable) is remain attached to the coat protein of filamentous phage. By applying this step, it may improve the protein folding as the phage may mimic missing constant domains of the original antibody [148].

Another approach to this problem is via fusing the scFv (single-chain fragment variable) to other proteins such as constant light chain domain, leucine zipper dimerization domain (ZIP) [149], Fc fragment (CH2 and CH3 domains) of mouse IgG1 [150], and alkaline phosphatase (AP) [151] to become more stable while retaining the functionality. According to Harper et al. [152], the fused scFv (single-chain fragment variable) is considered to direct to the configuration of dimers, which should have better avidity and stability compared to original monovalent scFv (single-chain fragment variable). Furthermore, in the case where scFv (single-chain fragment variable) fused to alkaline phosphatase (AP), there is the possibility of direct detection with substrate without the use of expensive antibody enzyme conjugates [56].

Besides diagnosis, the scFv (single-chain fragment variable) fusion proteins could also provide a valuable tool in controlling infectious disease, whereas phages displaying antibody fragments can be used for antigen detection and recognition in ELISA or immunoblot detection [88]. Moreover, the detection can be easily magnified by fusing a colour-generating enzyme such as alkaline phosphatase to the pVIII protein which was greater copy number compared to the pIII protein [115]. For instance, a sandwich-type ELISA (enzyme-linked immunosorbent assay) was developed by Kerschbaumer et al. [151] based on the scFv (single-chain fragment variable) fusion proteins in both capture and detection of a plant pathogen.

In addition to fusion protein, the new perspectives for exploiting scFv (single-chain fragment variable) have too widely opened with various fluorescent proteins, giving rise to fluobodies. Griep et al. [153] in their study successfully fused scFv (single-chain fragment variable) to a mutant of green fluorescence protein (GFP), followed by expression in *E. coli* and after that used it for direct labeling in flow cytometry and immunofluorescence experiments. This fluobodies do not fade after illumination in comparison to fluorochrome-fluorescein-isothiocyanate- (FITC-) conjugated antibodies. In a different report, scFv (single-chain fragment variable) antibodies were fused with different wavelengths of fluorescent proteins to permit concurrent multicoloured staining with antibodies against different antigens [154]. Alternatively, fluobodies were also utilized in a novel assay, called fluorophor-linked immunosorbent assay (FLISA) which is similar to ELISA. Theoretically, scFv (single-chain fragment variable) is fused to a fluorescent protein and antigen binding is detected by measuring the fluorescence. It thus make this assay faster and simpler than the comparable ELISA, and circumvent the need of secondary antibodies [155].

Overall, scFv (single-chain fragment variable) demonstrated to be valuable reagents in both detection and diagnostics. With new phage display antibody libraries, one can generate a range of scFv (single-chain fragment variable) specifically directed against any antigen economically, quickly, and without the bother of immunizing animals and manipulating hybridomas.

### 8.3. Potential Application of Single-Chain Variable Fragment (scFv) in Breast Cancer Studies

Breast cancer is a malignant tumor that has developed beginning from cells of the breast. A malignant tumor is a group of cancer cells that may attack adjacent tissues or spread (metastasize) to distant areas of the body. Nowadays, breast cancer is the most common and frequent cause of cancer-derived death in women [156]. There are conventional therapies such as surgery, chemotherapy, radiotherapy, antiestrogen therapy which are not able to eliminate occult cancer cells and therefore to prevent metastatic diseases, relapses, and bears the risk of side effects on non-tumor tissues [157]. Therefore, sensitive detection of residual cancer cells in breast tissue may have important therapeutic and prognostic implications. The potential targets for immunotherapy could be achieved by identification of tumorspecific or associated antigens on the surface of breast cancer cells [158]. The first promising approaches which have been published in immunotherapy involve the application of monoclonal antibodies, immunotoxins, bispecific antibodies, or vaccination with tumor-specific antigens [159, 160]. Until now, recombinant antibody fragments have been proven promising *in vitro *as well as in phase I and II clinical trials in certain types of cancer [161, 162]. Nevertheless, antigens suitable for immunotherapy in breast cancer are still rare.

A fusion between the lymphocytes of Balb/c mice sensitized with the MCF-7 breast carcinoma cell line with Sp2 myeloma cells [163]. From this study, the mouse hybridoma clone (C3A8) established was very stable in secreting monoclonal antibody. mAbs produced by C3A8 reacted very strongly to the human breast cancer cell lines MCF-7 and T47-D but showed negligible reactivity against other human cancer cell lines and series of normal tissues [163, 164]. The C3A8 clone showed 100% positivity after five limiting dilutions and secreted IgM Mab with kappa light chain. An immunohistological study performed by these researchers showed that the Mab reacted to lobular breast and fibroadenoma cancer tissues at the cytoplasmic region. While the reactivity of Mab C3A8 towards MCF-7 cell line was markedly reduced by trypsin but not to periodate or neuraminidase. Therefore, the epitope recognized by Mab C3A8 is not a carbohydrate or glycoprotein but an endopeptide consisting of arginine and lysine side chains.

Studies on the binding activities of monoclonal antibody to breast cancer and antigenic sites of the MCF-7 could be performed using the scFv (single-chain fragment variable) because it is highly immunogenic and has distinct antigens that elicit antibodies specific for this type of cancer [165–167]. For instance, in site-directed mutagenesis, the important variable regions or residues involved in binding activity could be determined and mutants with higher affinity can be created. This is particularly important for improvement of the performance of clinical assays for MCF-7 breast cancer cells. Besides, the smaller molecules of scFv (single-chain fragment variable) could as well facilitate crystallographic study of MCF-7 cells-antibody interaction. All these applications can be carried out with much ease on small molecules like scFv (single-chain fragment variable) than the intact IgM molecule. In addition, since the bacteriophages are highly immunogenic [117], mice immunized with the phage-displayed scFv (single-chain fragment variable) could produce anti-idiotypic antibodies which are required for receptor studies of MCF-7 breast cancer cells.

## 9. Conclusion

mAbs in scFv (single-chain fragment variable) format is preferred than intact antibodies due to its smaller size and less possibility of developing antimouse antibody response. Besides, it is possible to be designed as bispecific antibody to work as immunotoxin. It thus makes scFv (single-chain fragment variable) as the best candidate for medical, diagnostic and research applications. As mentioned in this review, range of antibodies fragments can be expressed in *E. coli *as active antigen-binding proteins. However, the levels of scFv (single-chain fragment variable) expression may widely vary. Therefore, several factors and parameters should be optimized to improve functional of each scFv (single-chain fragment variable) antibody expression as well as better antigen-binding affinity.

## Figures and Tables

**Table 1:** FDA-approved therapeutic antibodies [3].

Brand name	Antibody	Target	Source	Year	Indication
Orthoclone^®^	Muromonab-CD3	CD3	All rodent	1986	Transplantation rejection
ReoPro^TM^	Abciximab	GPIIb, IIIa	Chimeric	1994	High-risk angioplasty
Rituxan^TM^	Rituximab	CD20	Chimeric	1994	Non-Hodgkin's lymphoma, rheumatoid arthritis
Zenapax^®^	Daclizumab	CD25	Humanized	1997	Transplantation rejection
REMICADE^®^	Infliximab	TNF-a	Chimeric	1998	Crohn's disease
Simulect^®^	Basiliximab	CD25	Chimeric	1998	Transplantation rejection
Synagis^TM^	Palivizumab	RSV F protein	Humanized	1998	RSV infection
Herceptin^®^	Trastuzumab	HER-2	Humanized	1998	Breast cancer
Mylotarg^TM^	Gemtuzumab	CD33	Humanized	2000	Acute Myeloid Leukemia
Campath^®^	Alemtuzumab	CD52	Humanized	2001	Chronic lymphotic leucemia, T-cell lymphoma
Zevalin^®^	Ibritumomab tiuxetan	CD20	Murine—with yttrium-90 or indium-111	2002	Non-Hodgkin's lymphoma
HUMIRA^TM^	Adalimumab	TNF-a	Human	2002	Inflammatory diseases: mostly autoimmune disorders like rheumatoid arthritis, psoriadic arthritis, Morbus Chron
Bexxar^®^	Tositumomab	CD20	Murine: covalentely bound to Iodine 131	2003	Non-Hodgkin's lymphoma
Xolair^®^	Omalizumab	IgE	Humanized	2003	Severe (allergic) asthma
Avastin^TM^	Bevacizumab	VEGF	Humanized	2004	Metastatic colorectal cancer, nonsmall cell lung cancer, metastatic breast cancer
TYSABRI^®^	Natalizumab	*α*4 subunit of a4*β*1	Humanized	2004	Multiple Sclerosis, Chron's disease
Erbitux^TM^	Cetuximab	EGFR	Chimeric	2004	Colorectal cancer, head and neck cancer
Vectibix^TM^	Panitumumab	EGFR	Human	2006	Metastatic colorectal carcinoma
LUCENTIS^TM^	Ranibizumab	VEGF-A	Humanized Fab	2006	Wet macular degeneration
Soliris^®^	Eculizumab	CD59	Humanized	2007	Paroxysmal nocturnal hemoglobinuria
CIMZIA^®^	Certolizumab pegol	TNF-a	Humanized (Fab)	2008	Crohn's disease, rheumatoid arthritis
Simponi^TM^	Golimumab	TNF-a	Human	2009	Rheumatoid and psoriatic arthritis, active ankylosing spondylitis
